# Local Perspectives on Environmental Insecurity and Its Influence on Illegal Biodiversity Exploitation

**DOI:** 10.1371/journal.pone.0150337

**Published:** 2016-04-15

**Authors:** Meredith L. Gore, Michelle L. Lute, Jonah H. Ratsimbazafy, Andry Rajaonson

**Affiliations:** 1 Department of Fisheries & Wildlife, School of Criminal Justice, Michigan State University, East Lansing, Michigan, United States of America; 2 School of Natural Resources, University of Nebraska-Lincoln, Lincoln, Nebraska, United States of America; 3 Groupe d’Etude et de Recherche sur les Primates de Madagascar (GERP), Antananarivo, Madagascar; 4 Malagasy Biologist Association, Antananarivo, Madagascar; Cornell University College of Veterinary Medicine, UNITED STATES

## Abstract

Environmental insecurity is a source and outcome of biodiversity declines and social conflict. One challenge to scaling insecurity reduction policies is that empirical evidence about local attitudes is overwhelmingly missing. We set three objectives: determine how local people rank risk associated with different sources of environmental insecurity; assess perceptions of environmental insecurity, biodiversity exploitation, myths of nature and risk management preferences; and explore relationships between perceptions and biodiversity exploitation. We conducted interviews (N = 88) with residents of Madagascar’s Torotorofotsy Protected Area, 2014. Risk perceptions had a moderate effect on perceptions of environmental insecurity. We found no effects of environmental insecurity on biodiversity exploitation. Results offer one if not the first exploration of local perceptions of illegal biodiversity exploitation and environmental security. Local people’s perception of risk seriousness associated with illegal biodiversity exploitation such as lemur hunting (low overall) may not reflect perceptions of policy-makers (considered to be high). Discord is a key entry point for attention.

## Introduction

The global illegal wildlife trade has dramatically accelerated in the past decade although it has long been recognized as a serious threat to biodiversity conservation and livelihood preservation. Alongside other forms of biodiversity exploitation and environmental “crimes”—such as illegal commercial logging and illegal unreported and unregulated fishing—wildlife crime is politically destabilizing, subverts the rule of law, undermines sustainable development investments and generates monetary proceeds that fuel other organized crime and conflict [1,2]. In the 1990s, illegal exploitation was viewed as a key driver of biodiversity loss and addressed by the field of conservation biology alone. Today it is viewed as a cause and consequence of environmental insecurity and the purview of a broad array of public, private and nongovernmental sectors (see special issue 90:4 of s).

Environmental insecurity (i.e., the other side of the environmental security coin) is widely considered a source and outcome of biodiversity declines and social conflict [3,4]; the term is readily used in global environmental high politics (e.g., [5,6]) and progressively related to biodiversity and development policy. Definitions of environmental security vary [7], but all seem to include the common theme of whether or not people have enough food, water and natural resources to live. Having physical access to a reliable and healthy source of and ability to pay for natural resources are coupled with adaptive capacity, or the ability to recover from socio-ecological shocks (e.g., tsunami). The presence of a reliable government working to effectively protect natural resources also impacts resiliency and provides people with access to active and healthy lifestyles [8,9]. In terms of environmental change, security is often considered the condition when and where individuals and communities: (a) have the options necessary to end, mitigate or adapt to risks to their human, environmental and social rights; (b) have the capacity and freedom to exercise these options; and (c) actively participate in attaining these options [10]. Accordingly, environmental securitization involves sustainable use of natural resources and increased capacity to avoid or mitigate risk of experiencing negative consequences of environmental change [11]. Environmental insecurity can be considered the absence, deficit, or lack of environmental security.

There are many ways to think about managing risks and negative effects of change associated with environmental insecurity and biodiversity exploitation. There are also myriad processes for translating high-level policy directives to ground-level programs. Criminologists proposed a typology of conflicts producing negative environmental consequences and insecurity. Broadly, conflict-environment relationships can be over (1) natural resources possession (i.e., focused on access to, control over, or use of natural resources like forests), (2) declining resources (i.e., concerned with scarcity and broad degradation such as drought), (3) destroyed environments (i.e., resulting from armed conflict and other military activities such as the “war on drugs”) and (4) processes of natural resource extraction (i.e., concerned with methods, techniques and necessity of extraction, including the resource itself and infrastructure, like hydraulic fracturing) [12]. Programs can be designed depending on the nature of the human-environment relationship.

Since 2013, U.S. policy on illegal biodiversity exploitation has been structured to reduce risks through improved and enhanced enforcement, reduced consumer demand and increased diplomatic cooperation [13]. The 2014 London Conference on Illegal Wildlife Trade called for a range of actions including reversing the high profit and low risk nature of the wildlife crime equation. These and other risk-based appeals from the global community to resolve negative consequences for people and biodiversity emerging from illegal exploitation have resulted in, among other outcomes, new collaborations, strategies and laws. Most of these high-level risk-based solutions fall into three paradigms. First, some entail “incident-specific” risk management whereby authorities react to problems, acting to address specific incidents as they encounter them in real time through intelligence (e.g., two men on Thai Airlines flight TG-350 were apprehended by Pakistani customs officials with 145 kg of pangolin scales after authorities received a tip). Second, “community-based management” promotes engagement of local community members by risk managers to address problems according to and defined by local context (e.g., conservancy managers in Namibia’s Wapuro Conservancy engaged local members in participatory risk mapping of wildlife poaching locations and intensity within conservancy boundaries). Third, “problem oriented management” proactively focuses on resolving the root problem(s) of crime incidents in a structured and focused manner (e.g., Sumatran rhino protection units use situational crime prevention to address local provocations, excuses and opportunities enabling rhino poaching).

The above-mentioned approaches represent top-down tactics for resolving risks associated with environmental insecurity and illegal biodiversity exploitation. Here, illegal means against the rule of law, or the nationally codified rules of a nation. Framing illegal biodiversity exploitation as a form of environmental insecurity influences the questions that are asked, the research that is prioritized and the solutions and policies that are proposed [14]. In many ways hierarchical strategies are reasonable, given the international community is required to measure and demonstrate progress at a global level [1]. A practical challenge to scaling *down* international guidelines is that empirical evidence about local level or front-line attitudes is often missing. Thus, policies that are well-intentioned but do not take into account local variations in human dimensions such as attitudes or behaviors may collapse at a local scale and ultimately fail to reduce risk associated with environmental insecurity and biodiversity exploitation. Locally generated insight can uniquely inform policy assessment, implementation and evaluation; solutions that fail to account for local realities may prove ineffective or even counterproductive. For example, asking local people to monitor illegal activity but underestimating social norms protecting relatives from sanctions may protect neither the resource nor the broader communities who access it [15]. Information about local risk perceptions (i.e., intuitive judgments) associated with environmental insecurity and illegal biodiversity exploitation can augment the growing knowledge base of technical assessments of insecurity, exploitation and crime. Risk perceptions influence individuals’ behavioral preferences for risk management, attitudes about biodiversity and public trust in managers [16].

One bottom-up approach for considering local perceptions of management responses to biodiversity exploitation and insecurity risks comes from cultural theory [17]. Cultural theory suggests four typologies of risk management responses based on cultural attributes (e.g., whether a society is egalitarian, fatalistic, hierarchical, or individualistic). One embraces risk as an opportunity for innovation and benefit, resulting in deliberate and purposeful counteraction. A second intentionally disregards or fails to consider risks in order to eliminate them. Societies may alternatively respond to risk by modifying existing regulations in an effort to reduce risks to lower levels. Finally, populations may dismiss or repudiate risks through pursuit of the status quo [17]. Preferences for risk responses may be in part determined by cultural conceptualizations of the fundamental characteristics of “nature” (i.e., the environment) often referred to as “myths of nature” [18] (e.g., beliefs in nature as fragile or resilient). Characterizing perceptions of the appropriate risk management-based response to environmental insecurity, regardless of the source of such conflict, deepens insight about how local people may respond to policy, technology, strategies or tactics [19]. Empirical evidence about local responses to policy can help predict buy-in for current and future risk management strategies (e.g., incident-based, community-based, problem-oriented). To this end, we set three objectives: (1) determine how local people rank the relative risk associated with current and future sources of environmental insecurity, (2) assess local perceptions of environmental insecurity, illegal biodiversity exploitation, myths of nature and preferences for risk management responses and (3) explore relationships between perceptions about environmental insecurity, myths of nature and risk perceptions on illegal biodiversity exploitation.

## Materials and Methods

### Ethics Statement

Michigan State University’s Committee on Research Involving Human Subjects IRB# x10-394 reviewed and approved all methods and procedures used in this research. A committee-approved informed consent was obtained in verbal form due to potential participant illiteracy. In instances where participants approved use of a digital voice recorder, consent was documented digitally. In all instances, participants had to verbally consent to participate in the study before data collection commenced.

### Data Archiving

Data are archived at: http://datadryad.org, [doi:10.5061/dryad.5q27k].

### Study Site

As one of the world’s hottest biodiversity hotspots with 75% of species being endemic (i.e., found no place else on Earth), Madagascar’s flora and fauna face numerous anthropogenic risks such as illegal logging and unsustainable commercial hunting [20, 21]. Three-quarters of Malagasy live in poverty, the literacy rate is 64%, 35% have access to an improved water source and the Gross National Income of $420 USD/year ranks Madagascar 178 of 184 nations [22]. Madagascar has been rated as having extremely high rates of environmental exploitation and natural resource trafficking, particularly wildlife and timber products [23,21]. Considering its relatively small land area compared to other countries, it is remarkable that Madagascar is estimated to supply 2.5% of the world’s live reptile trade and has one of the highest rated volumes of illegal trade in the world [24].

Illegal natural resources exploitation and wildlife trafficking in Madagascar are at least in part a function of extremely rare species in high demand, corruption, high poverty rates, poor governance and prolific natural resources [25, 26]. In particular, especially since experiencing a political crisis in 2009, Madagascar has experienced an upsurge in magnitude and extent of illegal harvesting of endangered hardwoods [e.g., rosewood (*Dalbergia spp*.)] and wildlife trafficking for the global pet trade [e.g., ploughshare tortoise (*Astrochelys yniphora*)] [27]. Transparency International’s 2014 Corruption Perception Index gave Madagascar a 28/100, ranking it 133 out of 175 countries; the 2013 Global Corruption Barometer survey reported only 2% of the Malagasy population sampled in a national survey felt the level of corruption had decreased a lot over time [28].

These trends paint a bleak picture for Madagascar’s degraded environments [29] and the people whose well-being depend on it [23]. Because many of the wildlife involved in trafficking originate in biodiversity-rich developing countries such as Madagascar, insights from research conducted there may have practical implications for other biodiversity hotspots around the world. In Madagascar and elsewhere, poaching can be a highly localized activity that directly feeds into the global wildlife trafficking supply chain.

Reducing risks to biodiversity and local livelihoods from wildlife trafficking in Madagascar, and elsewhere, is a high policy priority [21]. Various institutions address these and other conservation risks in Madagascar, including the *fokonolona*, or a group of people living within the same village or Malagasy indigenous community [30]. Fokonolona may, for example, create and enforce rules and sanction offenders independently from, but also in concert with the federal government. Here, illegality may not refer only to the rule of law but also the rules in use, or the normative guidelines locally recognized and administered by the fokonolona. Madagascar also has provincial and federal-level protected areas that create and enforce wildlife trafficking rules. In reality, conservation-related rules are enforced inconsistently and most biodiversity exploitation occurs overtly [31].

**Fig 1:**
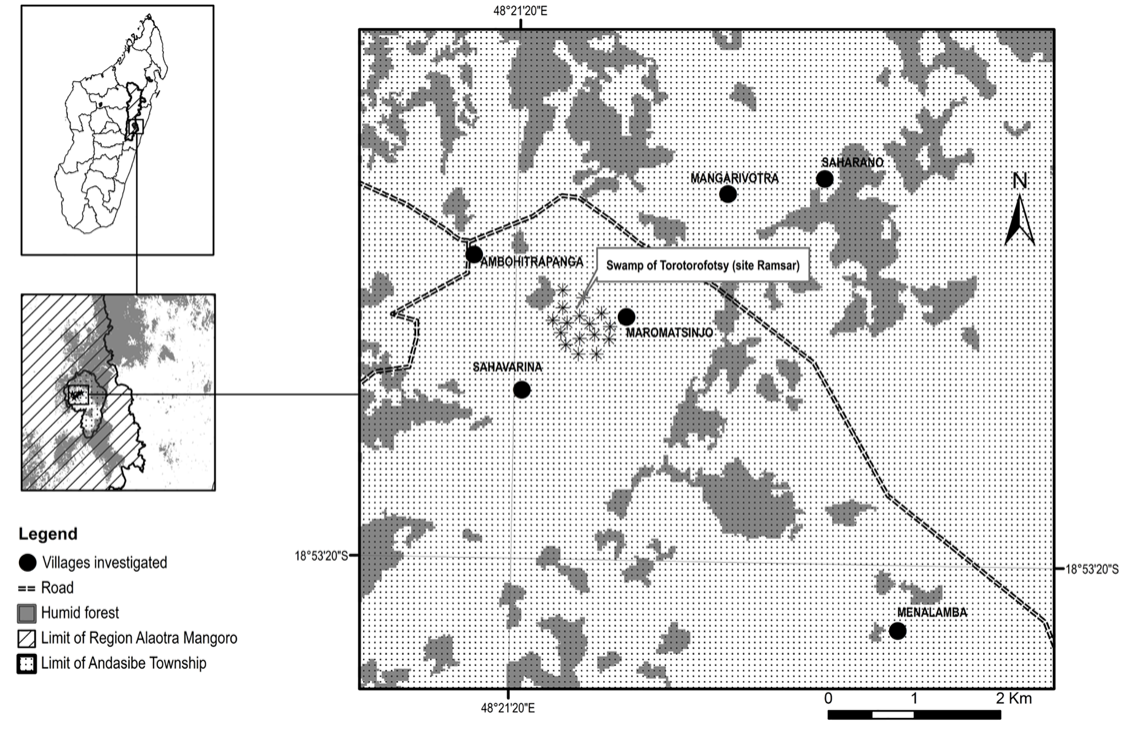
Map of Torotorofotsy Protected Area, our study site within east central Madagascar. Points denote 11 villages visited for interviews. Participants from two villages (Andasitsimanga and Menalamba Analakely) were interviewed in Menalamba village. Menalamba, denoted by a single point on the map, encompasses four smaller subvillages of a larger jurisdictional village that were visited on separate days.

Our research occurred within and adjacent to the Torotorofotsy Protected Area of east central Madagascar (Fig 1). Torotorofotsy is one of nine Ramsar sites (i.e., the oldest modern global intergovernmental environmental agreement) in Madagascar, comprised of near-natural permanent and temporary marshes, primary rainforests and secondary forests fragmented by agricultural zones. Endangered species supported in the area include the Golden Mantella frog (*Mantilla aurantiaca*), Yellow Mantella frog *(Mantilla crocea)*, Slender-billed flufftail (*Sarothrura watersi*), Meller’s duck (*Anas melleri*), Serpent eagle (*Eutriorchis astur*), Madagascar grass owl (*Tvto soumagnei*) and four endangered species of lemur. This location has a variety of ongoing biodiversity exploitation activities including illegal lemur poaching for bushmeat and pet trade, illegal logging and illegal charcoal production. In this context, these activities are illegal in that they are against the rule of Malagasy law. Torotorofotsy has a number of local anthropogenic activities that effect the local people and environment such as ecotourism, mining, siltation of marshland from deforestation and *tavy* (i.e., swidden agriculture).

### Community Entry

We collaborated with a guide from a Malagasy non-governmental organization to facilitate community introductions and community entry of the field research team (i.e., two male Malagasy, one female American) to Taratra, the local Vondron’Olona Ifotony (VOI). VOIs are community-based groups responsible for natural resource management. Before data collection commenced, the field team and guide attended a VOI meeting not related to this research. VOI representatives helped identify villages meeting the following criteria where the field team could commence research: (1) within or adjacent to Torotofotsy, (2) had a VOI representative present and available to be introduced to the team, (3) were from a village reasonably accessible by 4x4 vehicle and one day’s drive from the town of Andasibe and (4) had a large enough population (i.e., ≥3 families) to warrant sampling effort within a short period of time. The field team and VIO representatives collectively agreed upon calendar dates to visit respective villages and commence interviews. In some instances, the NGO guide, VOI and field team prearranged meeting locations so that participants from more remote villages could volunteer to participate in the research. The group had a discussion about not incentivizing participation in the research (i.e., paying a monetary compensation to participants). VOI representatives communicated to all participating village leaders that there would be no compensation for participating in the research but that researchers would deliver research findings back to them along with local and national decision-makers as soon as possible. VOI representatives were asked by the research team to explicitly emphasize informed consent (i.e., anonymity, the voluntary nature of participation). Each VOI representative was responsible for communicating information to all persons 18 years or older in his village, including the date interviews would be conducted and that participation was voluntary. Given Malagasy *fomba* (i.e., customs related to respect for elders’ guidance), the research team negotiated a nominal compensation (US$2.50) for the VOI representatives’ efforts.

### Measurement

We used face-to-face structured interviews to achieve objectives; we measured perceptions of environmental insecurity (focused on whether or not people have food, water and natural resources to live), risk perception (focused on intuitive judgments about costs and benefits), risk management responses and forest-related activities related to illegal biodiversity exploitation (i.e., [live or dead] lemur hunting, charcoaling, logging). Local definitions of illegality may differ from formal international or state definitions; for this research we were not interested in delineating the rule of law from rules in use and so did not distinguish between the two during data collection. The interview instrument (Fig A in S1 File) was developed in English, translated into Malagasy, verbally-back translated into English and conducted in Malagasy. This approach has worked successfully for us in the past in similar research contexts (e.g., [32]). Most questions were easily translated into Malagasy. When words were unclear (e.g., politics, “better off”) the American field researcher provided more specific definitions and synonyms, the two Malagasy researchers offered ideas and all three reached an agreement on the appropriate word choice for the interview. Conceptual triangulation was achieved through team decision-making to ensure interview clarity and cross-checks of translation [33]. The field team debriefed each evening after interviews concluded, continuously reevaluating the consistency of methods to maintain reliability and validity [34]. The team used the same protocol to minimize the potential for interviewer bias that might occur through inconsistent order or wording of questions [35,36]. A three-year history of research collaboration among the field research team aided the validity and reliability of data collection along with use of visual scales and aids [32].

A substantial body of literature addresses methodological issues associated with data collection associated with sensitive questions (see [37] and multiple articles within the special issue of Biological Conservation focused on detecting and understanding non-compliance with conservation rules). We minimized the potential to experience response bias to sensitive questions by using established techniques such as not anchoring questions directly on the respondent and dedicating time during the interview to inform participants that we were not interested in their personal behavior that may be illegal, but rather knowledge of *other* people’s behavior that may be illegal (Fig A in S1 File). Given our collective experience exploring biodiversity exploitation in this and other regions of Madagascar, we are also aware that a large proportion of biodiversity exploitation is overt and therefore less sensitive than other illegal behaviors that may be considered covert, such as property theft.

Verbal informed consent was obtained before interviews commenced. After an icebreaker question involving food preferences, participants were asked to rank order environmental risks using laminated photo cards (e.g., photograph of an eroding hillside and the corresponding Malagasy word, lavaka) (Table 1). All subsequent questions had 4-point Likert-type response options. Visual scales and aids were used to assist interviewees in conceptualizing the 4-point scale [32].

**Table 1:** Eight threats to environmental security in Torotorofotsy, Madagascar in May 2014 (n = 88).

Environmental Risk (in English)	Malagasy Translation	Mean (SD) Range 1–8
Cyclone	Rivodoza	6.82 (1.52)
Mosquitos/malaria	Moka	5.87 (1.87)
Forest fire	Doro-tanety	5.14 (2.08)
Swidden agriculture	Tavy	4.63 (1.95)
Erosion	Lavaka	4.60 (2.03)
Poaching	Fitrandrahana tsy ara dalana	4.22 (2.02)
Illegal logging	Kapakapa	4.15 (1.90)
Development	Fandrosoana	3.76 (2.71)

Each threat was visually depicted to participants using a laminated photograph labeled with the Malagasy word or phrase. Cyclones and malaria were the highest ranked risks and development the lowest ranked.

### Sampling

The field team opportunistically sampled individuals 18 years or older living within 11 villages between May 27-June 5, 2014. Within each village, a male VOI representative communicated in person to all village households and posted an announcement in writing in a central meeting place that a team of Malagasy and American researchers were funded by a U.S.-based university and interested in interviewing men and women willing to share opinions about activities affecting the local environment. Ultimately, interviewees were self-selecting. Both Malagasy researchers conducted interviews simultaneously with the American researcher alternating attendance and observations between Malagasy researchers.

Because we were interested in exploring conceptual ideas and not generalizing results to the entire Malagasy population, we did not use a probability sample to collect data. Opportunity sampling is germane when researchers are looking to gain access to potentially difficult data sources (e.g., geographically hard to access groups of people) and have a limited timeframe with which to collect data. The technique relies on the local knowledge and attributes of the researcher to identify a sample. We minimized threats to external validity (i.e., generalizability of results) by not connecting results to the broader population or claiming representativeness of results. We maximized internal validity (i.e., truthfulness of results) by asking multiple questions about the same concept and calculating reliability coefficients using software [38].

### Data Analysis

We used STATA to analyze data (v. 13.1, StataCorp, College Station, TX, USA) and parametric statistics because our tests for skewness and kurtosis fell within boundaries of a normal distribution. Scaled items were evaluated for internal consistency using Cronbach’s alpha [39]. Environmental insecurity consisted of 10 items averaged (ranging 0–3; α = 0.72) and risk perception consisted of 3 items averaged (ranging 0–3; α = 0.77) (Table A in S1 File). We used ordered logit regression to explore relationships between ordinal variables that met the parallel lines assumption: environmental insecurity, myths of nature and risk perceptions on illegal biodiversity exploitation behaviors [39–41].

## Results

We interviewed 88 participants from 11 villages and encountered four response refusals, indicating participants did not feel pressured to respond to participate or answer potentially sensitive questions. The majority of participants were female, ranged from 18 to 80 years old and had an average of four children in the household; most individuals were Betsimisaraka although participants reported identifying with at least five of Madagascar’s 18 recognized tribes (Table 2; [42]).

**Table 2:** Descriptive statistics based on interviews (n = 88) with Torotorofotsy, Madagascar residents, May 2014.

Characteristic	Statistic
Female	65%
Male	35%
Age	M = 39 (range: 18–80 years)
Number of children	M = 4 (range: 0–12 people)
Number of siblings	M = 6 (range: 1–13 people)
Years lived in area	M = 18 (range 1–63 years)

Five ethnic groups were represented in the sample” 59% Betsimisaraka, 15% Merina, 22% Bazanozano, 3% Shihanaka, 1% Antaemoro.

Our first objective explored how participants ranked relative risks associated with eight current and future sources of environmental insecurity risks. Cyclones were ranked most and urban development least risky sources of environmental insecurity (Table 1).

**Fig 2:**
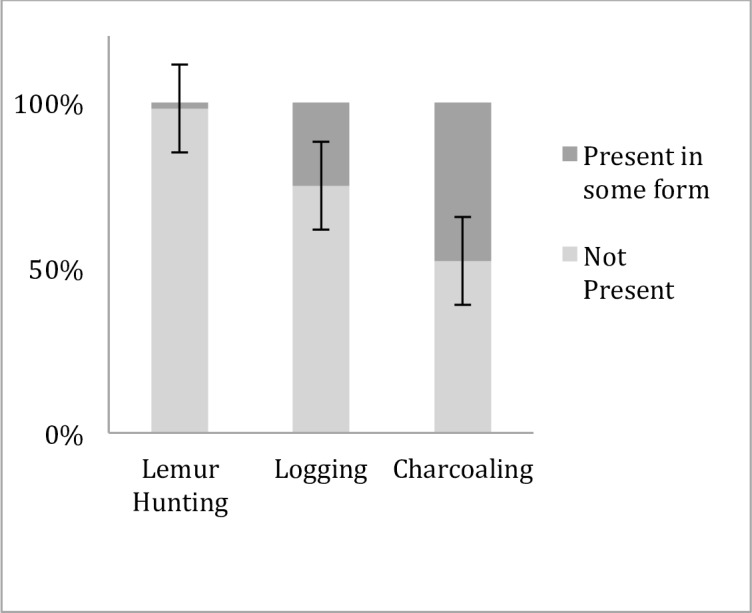
Local perceptions of illegal biodiversity exploitation rates. Error bars denote standard error. All activities are illegal under the rule of law in Madagascar.

Our second objective assessed local perceptions of environmental insecurity, illegal biodiversity exploitation, myths of nature and preferences for risk management response. All participants acknowledged some level of environmental insecurity although the average level of insecurity perceptions was 2.19 (range: 0–4; SD = 0.62). Approximately half of study participants acknowledged some level of local biodiversity exploitation in the forms of illegal timber and charcoal production (Fig 2).

**Fig 3:**
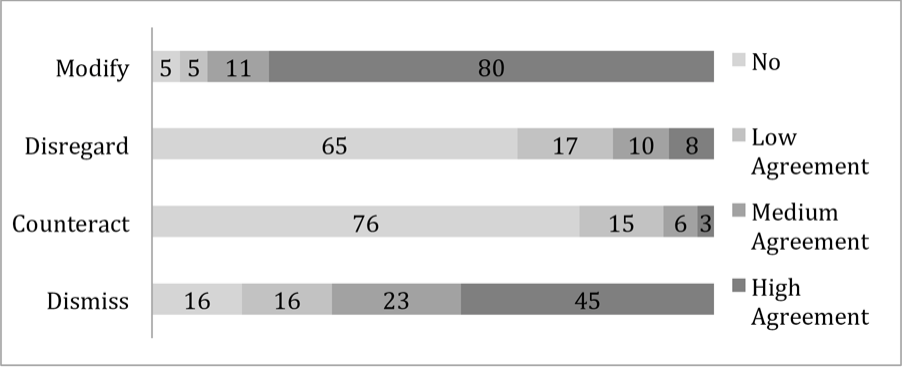
Proportion of participants (n = 88) agreeing with four risk management responses to environmental insecurity, Torotorofotsy, Madagascar, May 2014.

Fewer participants acknowledged the presence of lemur poaching, which is against the Malagasy rule of law. We did not ask direct questions concerning how much a participant personally engaged in the activity so as to avoid self-incrimination and social desirability bias [38], but rather asked about the level of activity in the general area. Majorities agreed that nature is random (67%, n = 59) and disagreed that nature returns to a balance (51%, n = 45). The majority of participants agreed environmental risks are unacceptable and need to be stopped (i.e., modify; 90%, n = 80). Majorities accepted there are environmental risks and the justifications to manage those risks (i.e., dismiss; 68%, n = 60), disagreed that environmental risks create new opportunities for creativity, innovation and development (i.e., counteract; 76%, n = 67) and that risks should be ignored (i.e., disregard; 64%, n = 56). The belief that nature returns to a balance had a negative effect on risk perception (B = -0.24, SE = 0.10, p<0.05), environmental insecurity (B = -0.18, SE = 0.07, p<0.01) and the preferred response to stop risks (i.e., modify; B = -0.19, SE = 0.08, p<0.05). The belief that nature is random had a negative effect on environmental insecurity (B = -0.20, SE = 0.04, p<0.0001) (Fig 3).

Finally, we explored the influences of environmental insecurity, myths of nature and risk perceptions on illegal biodiversity exploitation behaviors (i.e., charcoaling, lemur hunting, logging). Among study participants, we found no significant relationship between environmental insecurity and risk perception. Ordered logistic regression revealed a positive relationship between risk perception and biodiversity exploitation via logging (B = 0.95, SE = 0.28, p<0.01). Risk perception and charcoal production were negatively related (B = -0.47, SE = 0.20, p<0.05). The belief that nature is random had a negative effect on environmental insecurity (B = -0.67, SE = 0.16, p<0.0001). The belief that nature returns to a balance had a negative effect on risk perception (B = -0.34, SE = 0.15, p<0.05).

## Discussion

Reducing risks associated with illegal biodiversity exploitation is a high global policy priority attracting global news media attention. The risk of extinction via illegal commercial poaching is real [43]. The risks to humans associated with illegal biodiversity exploitation are no less severe [15]. It is understandable that diverse sectors are forming public-private partnerships to arrest, direct funding to, support research on and redeploy resources to confront exploitation. High-level policies supporting risk reduction are timely and appropriate; increasing the probability that such policies are effective and efficient at achieving objectives is paramount because many risk outcomes are irreversible. The principle of efficacy requires policies be able to scale down. Below, we discuss the primary implications of our research to this principle and pose questions for future research.

In some ways participants’ risk rankings appear to reflect the extant literature on local people’s perceptions of risk and social psychological factors influencing environmental risk perception. We know individuals tend to perceive risks as being higher or greater when they are generally considered to be involuntary, catastrophic in nature and scale, severe, worrying or unfamiliar (e.g., [44,45]). It is unsurprising then that cyclones, which in Madagascar have the potential to inflict substantial devastation on local people, environments and the government, were rated as most and urban development ranked least risky. What is noteworthy from our results however, is that local people’s perception of risk seriousness associated with illegal biodiversity exploitation such as lemur hunting (which was low overall) may not be reflexive with perceptions of policy-makers at national or international levels (who generally consider exploitation to be high) (e.g., [21]). When public perceptions of risk are not reflective of technical risk assessments, barriers to effective risk management can emerge, such as policy opposition or non-compliance [46,47]. Such discord is a key entry point for communication, outreach, or educational specialists to promote reflexivity.

A vibrant debate over how to attend to illegal commercial wildlife trafficking and other risks associated with global environmental conflicts is ongoing; some argue associating illegal biodiversity exploitation with environmental insecurity problematically limits response options to those within the militaristic sphere and hampers exercise of justice (e.g., 7) because borderlands become the site of security interventions [48]. In particular, connections between biodiversity declines and violent conflict seem to guide discussions about the extent to which the risks posed by degradation constitute insecurity or not. Others posit that environmental security discourse helps elevate biodiversity exploitation to high politics, resulting in the necessary political awareness and sense of urgency required to resolve environmental problems [6]. Regardless of the position or conclusion of this interesting set of publications, empirical evidence of local perceptions appears to be lacking. Thus the extent to which *any* of these discussions can wholly inform assessment of the responsiveness of policy to local needs and contexts is questionable.

Results herein offer, to our knowledge, one if not the first exploration of local perceptions of illegal biodiversity exploitation and environmental insecurity. Data challenge certain assumptions and validate others. Simple regression analysis helped us consider participant perceptions about precursors to different types of illegal biodiversity exploitation. Among our study participants, if the policy objective is to reduce or mitigate charcoal production in a protected area, it is essential to focus on the psychological aspect of associated risk perception as opposed to the socio-environmental dimensions of environmental insecurity such as access to land to grow food or a reliable source of clean drinking water. It may be the case that broader, large-scale socio-environmental processes and influences were less salient in the minds of our study participants. Indeed, others have found gaps in understanding the causes and consequences of climate change from populations vulnerable to drought associated with climate change [9]. Among our sample, the cognitions underlying risk perceptions associated with charcoal production are the key antecedents to human behavior that can be targeted by interventions involving, for example, education, communication, social marketing or regulatory changes.

We found myths of nature influenced environmental insecurity. Our results suggest that individuals who do not believe nature to be resilient have higher risk perceptions and perceptions of environmental insecurity. These same individuals may be less likely to accept risks and demand counteraction from managers. Interestingly, findings also point to greater perceptions of environmental insecurity and perceptions of rates of biodiversity exploitation via logging for those that believe nature to be random. Culturally-generated beliefs about nature can have important implications for preferred risk responses and overall goals of nature management and biodiversity conservation. If nature, and thus environmental change, is culturally considered to be random, environmental risks and certain exploitive activities may be more acceptable. Alternatively, if nature is considered fragile, environmental risks may undermine environmental security and tolerance for biodiversity exploitation. Although our study is limited in its ability to model all involved concepts, results suggest further exploration of the relationships between local perceptions of risk and risk response, environmental insecurity and biodiversity exploitation would be fruitful because these cognitions influence behavior. If behavior change, such as compliance, is a desired policy outcome, attitudes must be considered [49]. Conservation psychology suggests that sometimes cognitions exert influence on behavior vis a vis a stepwise process in someone’s mind (i.e., attitudes influence behavior, attitude change precedes behavior change, and information is needed to amend attitudes). For example, if an individual has high perceptions of risk, those high perceptions are more likely to lead to greater feelings of environmental insecurity which in turn results in greater participation in some illegal biodiversity exploitation activity.

Some security experts recognize threats to security of the homeland and environment differ greatly by degrees of intention and levels of violence [50]. These differences may help explain variation in individuals’ perceptions about the relationship between insecurity and biodiversity exploitation. Local perceptions can clearly function as an important human dimension within the biodiversity exploitation equation. Ignoring these human dimensions may complicate efforts to garner support for risk management or other policies designed to reduce negative effects of risk. Within our study context, the inconsistent manner with which environmental laws are reinforced in Madagascar and the involvement of communities in managing local natural resources complicates matters. If we had parsed out the difference between breaking the rule of law versus the rules in use, it is possible results would be different; future research exploring these differences would have practical implications for enforcement and other compliance activities.

A final confounding factor is the possibility that when some forms of illegal biodiversity exploitation are being perceived as threats to security in general, there is the danger that the citizens of one country (e.g., within Madagascar) will resent exploitation from other countries (e.g., outside Madagascar) more so than exploitation created by their fellow citizens [50]. This conservation-based “xenophobia” exists because conceptually, security is most often understood in the geopolitical terms of containment and exclusion [7]. One outcome of such nationalism is that local people may not perceive a sense of responsibility or culpability and cognitively justify their or their own family’s exploitive behavior.

Ultimately, all of these possibilities remain empirical questions and warrant additional research from the global environmental change community, particularly from the local perspective and potentially stratified by sociodemographics (i.e., gender, age, income). This is because changes explored at the globally and regionally averaged level can mask important local exceptions to general trends [51] that are essential for effective policy alternative selection, implementation and evaluation.

## Supporting Information

S1 File Supporting Information. Figure A. Interview instrument. Table A. New variables created for analysis.
(DOCX)
