# Supplementary material for: Local Perspectives on Environmental Insecurity and Its Influence on Illegal Biodiversity Exploitation
Source: PLoS One. 2016 Apr 15;11(4):e0150337. doi: 10.1371/journal.pone.0150337 (PMC4833313; doi:10.1371/journal.pone.0150337)
Supplement: S1 File — Figure A. Interview instrument. Table A. New variables created for analysis. (DOCX) [file pone.0150337.s001.docx]

S1 Fig A. Interview Instrument

*INTERVIEW NUMBER (ML TO FILL OUT): _ _ _*

**MADAGASCAR**

**MAY 2014**

“My name is ***FILL IN***.” I am a researcher working with “*MICHIGAN STATE UNIVERSITY (MSU)*.” I would like to talk to you about your opinions about the Malagasy environment because you live in an area of Madagascar that has a lot of wild animals and trees, some of which are used by people for their daily lives. Sometimes the environment is conserved as a park. I am trying to understand what local people think about the environment and human activities that can effect the environment. There are no wrong answers to the questions that I will ask. Your participation will help researchers understand the relationship between natural resource use and the lives of local people. Results from this study will be summarized in a report and presented to different groups that work help the lives of local people and wild animals.

I work at a university that sets rules about how I do my job. Some of these rules are that personal information such as your name will never be associated with your responses. Also, the information you share with me is private and under your control. I will only give your interview a number. I will take any information you are willing to share with me back to MSU and study the entire group of interviews I obtain, as opposed to individual interviews. The location of this village will not be named. The money for this study comes from my university, Michigan State University.

You may choose not to participate in the interview at any time. You may also choose to not answer a particular question of the interview. If you do not understand the questions please ask and I will be happy to explain in greater detail. You must be at least 18 of age to participate in this interview. By saying "Yes I understand" you are telling me that you are at least 18 years of age and want to participate. The entire interview should take about 40 minutes of your time. Do you have any questions before we get started?

**ICEBREAKER**

**Interviewer reads aloud: “This first set of questions are examples of the types of questions I will be using.”**

1. Please rank the following foods in order of least to most favorite, with least being #1 or on the bottom. There is no right or wrong answer

[ ] [ ] [ ] [ ]

a. Taramaso

b. Henomby

c. Trondro

d. Vorona

2. To what extent do you agree with the following question:

**(0 = do not agree at all/not relevant/won’t answer; 1 = a little; 2 = somewhat; 3 = a lot)**

My favorite color is blue? [0] [1] [2] [3]

**RISK PERCEPTION & RANKING**

**This question asks how certain risks compare to each other. Risks are hazards, threats, or chances. Environment is the natural world including nature, people, plants, animals, water, and all living things.**

3. Please rank the following risks in order of least to most threatening, with least threatening being #1 or on the bottom.

LOW RISK [ ] [ ] [ ] [ ] [ ] [ ] [ ] [ ] HIGH RISK

a. Rivodoza

b. Tavy

c. Fandrosoana

d. Lavaka

e. Dorotanety

f. Kapakapa

g. Moka

h. Fitrandrahana tsy ara dalana

**Read aloud: “These questions focus your judgments about risk. Please tell me the extent to which you agree with each statement. “**

*(0 = do not agree at all/not relevant/won’t answer/none; 1 = a little; 2 = somewhat; 3 = a lot)*

4. Gemstone mining poses harms the nature. [0] [1] [2] [3]

5. Lemur hunting poses harms to the nature. [0] [1] [2] [3]

6. Charcoal making harms the nature. [0] [1] [2] [3]

7. Cyclones pose harms to the nature. [0] [1] [2] [3]

8. Drought poses harms to the nature. [0] [1] [2] [3]

9. I worry about crime in my community. [0] [1] [2] [3]

10. The political crises posed harm to my community. [0] [1] [2] [3]

11. I worry about the effects of national politics on my community. [0] [1] [2] [3]

12. I worry about corruption in my community. [0] [1] [2] [3]

13. I worry about conflicts between social groups in my community. [0] [1] [2] [3]

14. I worry about either keeping my job or loosing my job. [0] [1] [2] [3]

15. I worry about becoming infected with HIV/AIDS. [0] [1] [2] [3]

**MALAGASY CULTURE AND SOCIETY**

**This set of questions asks your personal opinion about local Malagasy society and culture. Please tell me the extent to which you agree with each statement. Nature means the environment, the Earth, or the living world.**

*(0 = do not agree at all/not relevant/won’t answer/none; 1 = a little; 2 = somewhat; 3 = a lot)*

16. I would not participate in civil action groups. The ones in power do only allow what they like. [0] [1] [2] [3]

17. Important questions for our society should not be decided by experts but by the people. [0] [1] [2] [3]

18. A person is better off if she doesn't trust anyone. [0] [1] [2] [3]

19. In a family adults and children should have the same influence in decisions. [0] [1] [2] [3]

20. It is important to me that in the case of important decisions in my family that everyone is asked. [0] [1] [2] [3]

21. We have to accept the limits in our life if we want or not. [0] [1] [2] [3]

22. There is no use in doing things for other people--you only get it in the neck in the long run. [0] [1] [2] [3]

23. Institutions should be organized in a way that everybody can influence important decisions. [0] [1] [2] [3]

24. I don't join clubs of any kind. [0] [1] [2] [3]

25. It is important to preserve our customs and cultural heritage. [0] [1] [2] [3]

26. The freedom of the individual should not be limited for reasons of preventing crime. [0] [1] [2] [3]

27. My ideal job would be my own business. [0] [1] [2] [3]

28. The police should have the right to listen to private conversations when investigating crime. [0] [1] [2] [3]

29. When I have problems I solve them on my own. [0] [1] [2] [3]

30. I prefer tasks where I work something out on my own. [0] [1] [2] [3]

31. An intact family is the basis of a functioning society. [0] [1] [2] [3]

32. I prefer clear instruction from my superiors about what to do. [0] [1] [2] [3]

33. Order is probably unpopular but an important virtue. [0] [1] [2] [3]

**NATURE**

**These questions are focused on different ways of thinking about nature. Nature means the environment, the Earth, or the living world.**

*(0 = do not agree at all/not relevant/won’t answer/none; 1 = a little; 2 = somewhat; 3 = a lot)*

34. Nature is forgiving and always finds its way back to a balance. [0] [1] [2] [3]

35. Small changes made by people have very big impacts on nature. [0] [1] [2] [3]

36. Nature forgives events to a certain point. [0] [1] [2] [3]

37. Nature is random. [0] [1] [2] [3]

**FOOD SECURITY**

**This set of questions focus on food security, or the access by all people to enough good food for an active and healthy lifestyle.**

*(0 = do not agree at all/not relevant/won’t answer/none; 1 = a little; 2 = somewhat; 3 = a lot)*

38. I worry about having a reliable source of food. [0] [1] [2] [3]

39. I worry about having a reliable supply of food. [0] [1] [2] [3]

40. I worry about having physical access to food [0] [1] [2] [3]

41. I worry about being able to pay for food. [0] [1] [2] [3]

42. I worry about having access to healthy food. [0] [1] [2] [3]

43. How good is Madagascar at protecting food? [0] [1] [2] [3]

**ENVIRONMENTAL SECURITY**

**This set of questions focus on whether or not people have enough food, water, and natural resources to live***.*

*(0 = do not agree at all/not relevant/won’t answer/none; 1 = a little; 2 = somewhat; 3 = a lot)*

44. I worry about having a reliable source of water. [0] [1] [2] [3]

45. I worry about having a reliable source of natural resources to live. [0] [1] [2] [3]

46. I worry about having a reliable supply of drinking water. [0] [1] [2] [3]

47. I worry about having healthy land to grow food on. [0] [1] [2] [3]

48. I worry about how healthy the forests are. [0] [1] [2] [3]

49. I worry about how healthy the lakes and rivers are. [0] [1] [2] [3]

50. I worry about how healthy the ocean is. [0] [1] [2] [3]

51. I worry about my ability to recover from a cyclone. [0] [1] [2] [3]

52. I worry about my ability to recover from drought. [0] [1] [2] [3]

53. I have a reliable source of energy. [0] [1] [2] [3]

54. I have reliable protection from disease [0] [1] [2] [3]

55. I have a reliable federal government. [0] [1] [2] [3]

56. I have a reliable local government. [0] [1] [2] [3]

57. I have a reliable fokonolona. [0] [1] [2] [3]

58. I have reliable natural resources to live. [0] [1] [2] [3]

59. How good is Madagascar at protecting the nature?

[0] [1] [2] [3]

**ACTIVITIES**

**The following questions ask your opinion about how often the following activities happen in this area. I am not asking how much or whether YOU do these activities, simply how much you think these activities are happening here.**

*(0 = do not agree at all/not relevant/won’t answer/none; 1 = a little; 2 = some; 3 = a lot)*

60. How much lemur hunting happens here? [0] [1] [2] [3]

61. How much charcoal making happens here? [0] [1] [2] [3]

62. How much bat hunting happens here? [0] [1] [2] [3]

63. How much cutting trees for wood occurs here? [0] [1] [2] [3]

64. How common is it to take live wild animals to sell as pets at market? [0] [1] [2] [3]

65. How common is it to take dead animals to sell at market for tourism? [0] [1] [2] [3]

**RISK RESPONSE**

**Responsibles have many ways they can go about managing risks to the environment, or nature. These questions ask your opinion about different management reactions.**

*(0 = do not agree at all/not relevant/won’t answer/none; 1 = a little; 2 = some; 3 = a lot)*

66. I accept there are environmental risks and the justifications to manage those risks. [0] [1] [2] [3]

67. Environmental risks are unacceptable and need to be stopped. [0] [1] [2] [3]

68. I try not to know about environmental risks because there is nothing I can do about them. [0] [1] [2] [3]

69. Environmental risks create new opportunities for creativity, innovation, and development. [0] [1] [2] [3]

**CONCLUSION**

**These final questions focus on your background. This information will be private and will never be associated with your responses.**

70. Age: [ ]

71. Profession: [ ]

72. Years lived in area: [ ]

73. Ethnicity: [ ]

74. Sex: [0 = male] [1= female]

75. Number of children: 0-numer

76. Number of siblings: 0- number

77. American Researchers [0=None] [1= Lute]

78. Malagasy Researcher [0= Dani] [1= Andry, 2 = ________, 3 = ________, 4 = __________]

79. Date:___________________________________________

80. Township: ____________________________________

81. Verbal consent granted: [0 = No] [1= Yes]

Other: _________________________________________

S1 Table A. We created two new variables for analysis. Environmental security consisted of 10 averaged items and risk perception consisted of 3 averaged items.

| **New Variable** | **Averaged Reponses (variable name and number)** |
| --- | --- |
| Environmental Insecurity | wornr (Q45), worws (Q44), worland (Q47) worlake (Q49), worcycl (Q51), wordro (Q52), new_reldisease (Q54) new_refede (Q55), new_relocal (Q56), new_relnr (Q58) |
| Risk Perception | polrisk (Q10) polworry (Q11) corrupt (Q12) |
